# Understanding the Unique Electronic Properties of Nano Structures Using Photoemission Theory

**DOI:** 10.1038/srep17834

**Published:** 2015-12-04

**Authors:** Soonnam Kwon, Won Kook Choi

**Affiliations:** 1 Beamline Division Group of PAL-XFEL Project Headquarters, Pohang university of science and technology, 77 Cheongam-Ro, Nam-Gu, Pohang, Gyeongbuk, Korea 790-784; 2 Materials and Life Science Research Division, Korea Institute of Science and Technology (KIST), Hwarangno 14-gil 5, Sungbuk Gu, Seoul, Korea 136-791

## Abstract

Newly emerging experimental techniques such as nano-ARPES are expected to provide an opportunity to measure the electronic properties of nano-materials directly. However, the interpretation of the spectra is not simple because it must consider quantum mechanical effects related to the measurement process itself. Here, we demonstrate a novel approach that can overcome this problem by using an adequate simulation to corroborate the experimental results. *Ab initio* calculation on arbitrarily-shaped or chemically ornamented nano-structures is elaborately correlated to photoemission theory. This correlation can be directly exploited to interpret the experimental results. To test this method, a direct comparison was made between the calculation results and experimental results on highly-oriented pyrolytic graphite (HOPG). As a general extension, the unique electronic structures of nano-sized graphene oxide and features from the experimental result of black phosphorous (BP) are disclosed for the first time as supportive evidence of the usefulness of this method. This work pioneers an approach to intuitive and practical understanding of the electronic properties of nano-materials.

Large molecules with sizes up to several nano-meters are intermediate in size between small molecules and ordered crystals, and therefore have many interesting properties. The size effects include edge-structure-induced features and quantum confinement induced electron momentum broadening in the band structure^1^. Numerous *ab initio* calculations of finite-sized graphene have shown various peculiar electronic properties owing to defects inherent in it^2^,^3^. However, these calculations mostly concentrated on the initial state of the materials without considering the effects of the measurement process itself. Nano-size angle resolved photoemission spectroscopy (nano-ARPES) enables probing of the electronic properties of a nano-sized single grain^4^, but the experimental results of these materials are difficult to interpret due to quantum mechanical effects that occur during the measurement process. Therefore, experimental results should be corroborated by an adequate simulation that represents the real experimental processes.

For ordered crystalline surface and periodic adsorbates on crystalline surface, a dynamic theory of ARPES has been successfully developed^5^,^6^,^7^,^8^,^9^. For gaseous and small molecules, exact atomic photoemission (PES) calculation was exploited to obtain PES simulation for the whole molecule; the method is called independent atomic center approximations (IAC)^10^,^11^,^12^,^13^,^14^,^15^. Use of a plane wave approximation (PW) of final state in relation with the Fourier transform of the initial state is a useful simplification of the ARPES results for some materials, such as flat materials composed of the same element^16^,^17^. In contrast, IAC is easily applicable to any complex system, and explains ARPES results adequately^15^,^18^,^19^. IAC includes a realistic measurement process in the formalism; this process is neglected in other calculations that use PW as the final state. However IAC cannot explain the effects of scattered electrons by nearby atoms, because it considers only direct photoelectrons.

The present report attempts to determine the electronic structures of nano-sized materials by ARPES simulation that uses IAC performed on wavefunctions obtained from *ab initio* calculation. The validity of this method was confirmed by comparing simulation results of the nano-sized graphite single layer without orientation order, to the experimental ARPES results of highly-oriented pyrolytic graphite (HOPG). Then our method has also been applied to other 2D-layered materials such as nano-sized graphene, its oxides, and nano-flakes of black phosphorous (BP)^20^. The results prove that the molecular-like approach and use of IAC to analyze photoemission are appropriate for low-dimensional materials with nanometer size and disordered structure. This work may provide intuitive and practical understanding of 2D-layered van der Waals nano-materials such as transition-metal chalcogenides (MoS_2_, WSe_2_), boron nitride (BN), and BP.

To simulate HOPG as a molecule, an armchair-edged, hexagonally-symmetric polyaromatic hydrocarbon (PAH) with 762 carbon atoms was used as the model (Fig. 1). The *x* axis is defined to coincide with the ΓK direction and the *y* axis is perpendicular to *x* axis. The detailed geometry of the PAH is shown in Fig. 2a. All calculations of the geometry were performed using the Gaussian 09 package^21^. The geometry was fully optimized using PBEh1PBE/sto-3g level of theory^22^. The Kohn-Sham (K.-S.) energy was scaled to compensate for the approximation of electronic relaxation and correlation effects. In our calculation, we chose a compensation factor of 1.0002; this value was obtained by scaling the binding energy of calculated band structure to coincide with that of the experiment (Fig. 1b). Then, the overall energies are shifted to give a value of zero to the midpoint between highest occupied molecular orbital (HOMO) and lowest unoccupied molecular orbital (LUMO). Figure 1b shows the band structure along the k_//x_ direction of HOPG. HOPG was tilted on purpose by θ = 6° along the *x* axis (Fig. 1c). A 100 eV p-polarized photon was incident on yz plane and the angle between electron analyzer and photon was set to be 50° (Fig. 1c). Electron analyzer was located as shown in Fig. 1c. Therefore, the angle resolved measurements (α) were made parallel to *x* axis. To simulate randomly oriented nano-sized grains of HOPG, PES intensities were averaged over those of PAHs with 360 equally-spaced azimuthal angles (ϕ). Simulated results for −10° < *α* < 10° (Fig. 1a) show striking resemblance to experimental results despite the complex detection geometry. Complete simulation at high symmetric geometry (θ = 0°) is compared to corresponding experiments in the supplementary information.

However, our simulation did not consider multiple scattering effects; it only considered directly ejected photoelectrons. This neglect of the scattering effect may cause discrepancy from experimental results because HOPG is multi-layered, so multiple scattering of photoelectrons by nearby atoms is not negligible. Fortunately, for single layered nano-materials, such as those considered in this study, this effect is expected to be minimal.

ARPES simulations (Fig. 2) were conducted using hexagonally symmetric PAH molecules with zigzag- or armchair-edged structures. We followed the definition and the rule of indexing defined by Stein *et al.*^23^ The geometry of each series of molecules was fully optimized using PBEh1PBE/sto-3g level of theory^22^. The PES intensity map of HOMO (Fig. 2b) was shown along the k_x_ and k_y_ directions and the PES intensity versus binding energy map (Fig. 2d) was shown along the ΓM and ΓK directions. The first member of the zigzag series was coronene and that of armchair series was hexa-peri-hexabenzocoronene (HBC). The calculation results of the first series are analogous to those of ref. 24. As the number of carbons increases, the properties of the infinite-sized molecule (graphene) can be inferred by extrapolating each series^23^. The energy band of ideal graphene were calculated using equations (1) and (2)^1^.


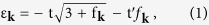



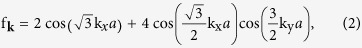


where ε_k_ is the energy of occupied lower (π) band of single layer graphene^1^ and k_j_ denotes the momentum of electrons in direction 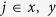, and *a* is the carbon-carbon distance. *t* and *t*’ are defined as hopping energies in ref. 1. In this study, they were used as parameters to perform curve fitting (Fig. 2d, red lines) of the simulated ARPES data points.

As the number of carbons increases, simulated PES intensity map approaches ideal band structure except for several distinct features, which appear to originate from edge structures. The most obvious feature is strong intensity along the lines that interconnect K points of the HOMO. This feature is overwhelmingly stronger in zigzag than in armchair structures. Zigzag edge structure tends to localize HOMO (Fermi level) electrons on the edge sites, whereas armchair edge structure does not show any edge confined pattern on the HOMO electron density; this result is consistent with previous reports^3^,^23^. We attribute the edge-induced ARPES feature to this peculiar behavior of HOMO electron distribution. If we ignore the contributions of the atoms near the edges during the ARPES simulation, the strong edge induced features disappear; this observation supports our hypothesis.

Another important feature related to nano-size is the quantum confinement effects. The PES intensity profile of HOMO along the ΓK symmetry line (Fig. 2c) shows a Gaussian distribution centered at each K point. Convolution using Gaussian distribution curves results in standard deviations σ (Fig. 2c, insets), which decrease as the size of PAH increases (Fig. 3, inset); the trends can be expressed as σ_k_ = 0.16 × *L*^−0.6^ Å^−1^ for zigzag structures and σ_k_ = 0.23 × *L*^−1.06^ Å^−1^ for armchair structures, where *L* is the smallest edge-to-edge distance of the nano-materials^25^. The relationship between the size of PAH and the broadening of band structure can be interpreted from the viewpoint of uncertainty principle as


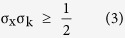


where σ_x_ can be expressed as a function of *L* and σ_x_ < *L*. To extract the dependence of the physical properties on size >5 nm, we extrapolated the values of energy gap and σ_k_ as a function of *L* using fitting functions obtained above. These values approached zero as *L* increased (Fig. 3).

As a next step, we investigate a system in which two epoxides are attached at antisymmetric positions on an armchair-edged PAH with 366 carbons (Fig. 4). The equilibrium geometry of the epoxide PAH was obtained after full geometry optimization using the PBEh1PBE/sto-3g level of theory^22^. The attached epoxide distorts the geometry of the PAH to a slightly curved surface. The total density of states (TDOS) of the occupied K.-S. energy levels are shown in the middle panel of Fig. 4a. HOMO was ≈−0.8 eV, which was not affected by epoxy adsorption. The projected density of states (PDOS) of O2p (Fig. 4a, bottom of middle panel) shows a single intense peak near −2.6 eV. This feature originates from the two oxygen atoms in the epoxide; the same feature can be also seen in the right panels of Fig. 4d, in which dispersion-less flat feature is obviously seen near −2.6 eV.

PES intensity maps of HOMO level (Fig. 4b) illustrate the dependence of PES on photon energy. In pristine PAHs, PES does not depend on photon energy; in epoxy ornamented PAHs, structure related patterns surrounding each K point strengthen as the photon energy increases. This enhancement of the pattern by increase in photon energy is evidence of the final state effect, which can be related to the wavelength of the emitted electron. For example, electrons with kinetic energy of 160 eV have wavelength λ  = 0.97 Å, which is smaller than the C-C bond length (1.42 Å). In this case, the structure factor (defined in the methods section) dominates the atomic factor. In contrast, for photon energy of 20 eV, the final state electron has λ  = 2.74 Å, which is much longer than the C-C bond length. In this regime, PES intensity is less influenced by the structure factor than in the regime with higher photon energy^13^.

Finally, the electronic structure of BP was elaborated using photoemission theory. Recently, ARPES experimental results on a bulk crystal of BP have been reported using s-polarized synchrotron radiation^26^. These results presented the opportunity to determine whether our method could qualitatively be applied to them. We modeled a molecule of a single-layered nano-flake of BP (Fig. 5a). Even though this molecule is different from that of ref. 26, we tried to compare the trends of the electronic structure; we believe that this comparison is analogous to the relationship of nano-graphene to a graphite single crystal. The geometry of this molecule was fully optimized using PBEh1PBE/sto-3g level of theory^22^. The K.-S. energy was scaled by 1.008 and the overall energy was shifted to +1.5 eV to best match the experiment^26^.

PES intensity of BP (Fig. 5b, upper panel) was simulated along high-symmetry directions, which correspond to those in ref. 26. The band structures along ZT’ (*x* axis) show striking resemblance between our simulation results and experiments. In contrast, along the ZL (*y* axis) direction, the simulation did not produce any significant features in the energy range from 0 to −2 eV, whereas in experiments clear band structure was observed in this region, although the intensity was extremely weak compared to the features in the other regions^26^. These peculiar properties have not been explained yet. We propose that they can be analyzed qualitatively using our approach as follows.

First, the characters of experimental band structures along ZT’ and ZL directions were assigned to 3p_y_ and 3p_x_, respectively (Fig. 5b, upper panel). From the phosphine 3p orbitals, significant intensity can be observed only under a special condition: photoemission from an atomic orbital that is parallel to the polarization of incident photon. Photoemission cross-sections of phosphine 3p_x_ (Fig. 5c middle) and 3p_y_ (Fig. 5c bottom) atomic orbitals by y-polarized 21.0 eV photons were calculated with electron emission along the ZT’ direction (XZ plane). The 3p_y_ orbital produces significant photoemission along the *x* axis, but the 3p_x_ orbital does not show any emission along the *x* axis (Fig. 5c). For the same reasoning, PES intensity from the 3p_z_ orbital along the *x* axis should be absent. Thus, along the ZT’ direction, only 3p_y_ contributes to the PES intensity. The same conclusion can be obtained for *x*-polarized photons in which incidence and emission are on the YZ plane. Along the ZL direction, only 3p_x_ atomic orbital contribute to the PES intensity. Except for the energy gap and the scale of energy levels, the overall features along ZT’ are reproduced by our calculation. The only fundamental difference is the dispersion-less feature at BE ≈−3.4 eV, which can be assigned to effect of edge structure. This feature disappears when the simulation ignores the contributions from edge atoms (Fig. 5a, red dotted rectangle).

Second, the absence of the band structure in the energy range from 0 to −2 eV along the ZL direction in our calculation and the extremely weak features in the same region of ref. 26 can be also explained if we consider the imperfection in s-polarization of the experimental setup. If we assume that an incomplete undulator geometry resulted in incomplete polarization of 99.9% along the *x* axis and 0.1% along the *y* or *z* axis, the simulation reproduces the low energy band structures (Fig. 5b, bottom panel), which are similar to those seen in the ref. 26. We assign these band structures to 3p_x_ or 3p_z_ orbitals. This explanation of the experimental result in ref. 26 is presented here for the first time.

In conclusion, we have explored the possibility of exploiting IAC approximation to simulate ARPES on nanometer-sized complex materials; IAC had been proven previously to be useful for small molecules. As expected from the theory in previous research, this study can explain the experimental results of various materials such as HOPG, graphene-like hexagonally symmetric PAHs, nano-sized graphene oxides, and black phosphorous. The methods described in this study can be also used to interpret experimental results complicated by disorder and diverse surface effects. In particular, the intricate final state kinetic energy dependent PES signal can also be interpreted for materials with complex geometries. The method introduced in this study can be used to visualize the selection rule by which initial band structures are probed for given polarization of photon and detection geometry. The method simplifies the task of interpreting results of experiments on nano-materials.

## Methods

ARPES experiments were conducted under ultra-high vacuum (2.0 × 10^−10^ Torr) using a high-resolution electron analyzer, VG Scienta SES 2002 with a 2D-CCD detector at the 8A2 undulator beam line of the Pohang Accelerator Laboratory (PAL) in Korea. Spectra of the valence bands were obtained using linearly-polarized photons with energies ranging from 100 eV to 400 eV. All the experiments were performed at room temperature. The HOPG crystal was cleaved outside the vacuum chamber using scotch tape and introduced into the chamber immediately. The sample was degassed at 400 °C for > 5 h to remove any adsorbed molecules. The cleanness of the sample was confirmed by measurements of the core levels of nitrogen, oxygen, and carbon.

PES intensity is measured at a specific angle and energy (*k*_f_, and *E*_kf_, respectively) from an initial state of ψ_i_, and can be expressed as


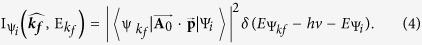


If we assume the final state as a plane wave (PW), equation (4) can be simplified to


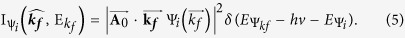


However, the PW approximation does not account for the dipole transition probability properly^18^. Therefore, to properly consider the photoemission process, the final state must be treated carefully.

IAC has been widely accepted in photoemission theory. IAC is based on a model in which emission from individual atomic centers of molecule occurs independently or coherently^13^. First, photoemission from individual atoms is obtained; the result corresponds to the atomic factor. Then the atomic factor is weighted by a structure factor and coefficient of the atomic orbital (explained in detail below), then summed to produce total photoemission intensity from the molecule. The structure factor contains phase difference due to the differences in propagation paths, which are caused by different distances of each atom from the center of a molecule. IAC can be expressed as


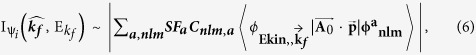


where *a* is the index of atom in a molecule, *n*, *l*, and *m* are the quantum numbers of the atomic orbital, *SF*_*a*_ denotes structure factor, and *C*_*nlm,a*_ is a coefficient of an atomic orbital, which will be explained in detail below.

The atomic factor is obtained by calculating the matrix element of 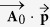 between initial and final states. The initial state is an atomic orbital with principal *n*, angular *l*, and magnetic quantum number *m*. The final state is a continuum orbital and the radial parts of it, *R*_Ekin,l’_ (r), can be obtained by solving a Schrödinger equation in which potential energy is the effective atomic central potential and kinetic energy is *E*_kin_. As a result, the solution is not a simple plane wave, but can be expressed as^19^


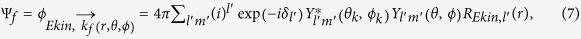


where *l*’ = *l* + 1, or *l*−1 because of the selection rule during photon-induced transition, so the partial wave expansion. *δ*_l’_ is the overall phase shift by the atomic potential. *θ*_k_, and *Φ*_k_ represent the polar and azimuthal angles of the emitted electron respectively.

After well-established analytic quantum mechanical treatments, equation (4) can be solved under the IAC approximation^13^. The PES cross section of the *n*th Kohn-Sham energy level can be defined as


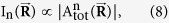



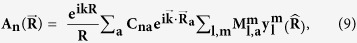


where 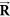 and 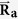 represent position vectors of the detector and of the *a*th atom in a molecule with respect to the origin of the molecule, respectively. 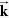 denotes the momentum vector of a photo-emitted electron. 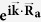 is a structure factor, and 
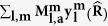
 is an atomic factor that represents the PES probability from the *a*th atom, and can be written as


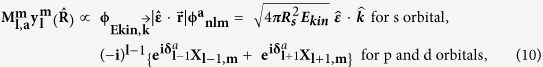


where *R*_*s*_ is the radial dipole matrix element of s orbital, 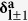 is the overall phase shift of the final state by the atom indexed by *a*, and 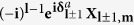 is the dipole transition matrix element of 
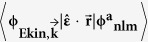
 for initial and final state angular quantum numbers of *l* and *l* ± 1, respectively. To calculate the matrix element, 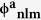
was replaced by atomic orbitals following the work of Grobman^13^. The complete expression of *X*_*l*±*1,m*_ can be found in table 5 of ref. 19, in which the information of polarization of incident photon, atomic orbital of initial state, and the detection geometry are presented. *C*_*na*_ represents the coefficients of the *a*th atom to the *n*th molecular orbital wave function, which can be obtained from density functional theory (DFT) calculation. More detailed description of the calculation methods can be found in the supplementary information and references therein. The photoemission intensity can be obtained as a function of momentum vectors of emitted electrons following equations (8)–(10), 11, 17.

## Additional Information

**How to cite this article**: Kwon, S. and Kook Choi, W. Understanding the Unique Electronic Properties of Nano Structures Using Photoemission Theory. *Sci. Rep.* **5**, 17834; doi: 10.1038/srep17834 (2015).

## Supplementary Material

Supplementary Information

## Figures and Tables

**Figure 1:**
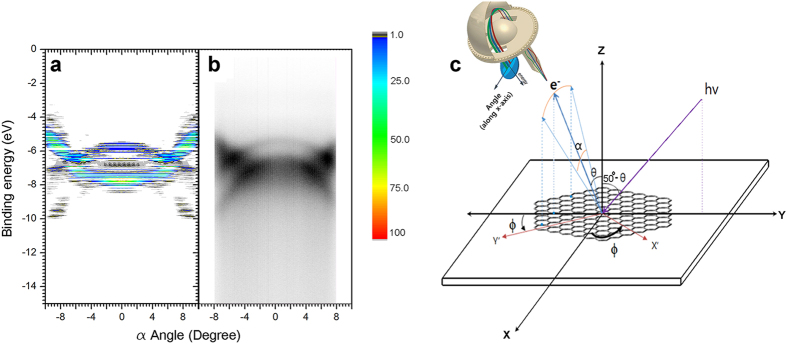
Comparison between the calculated and experimental ARPES intensity map for HOPG with tilt angle, θ  =  6°. (**a**) Calculation (**b)** Experiment. (**c)** Definition of geometrical parameters used for experiment and calculation of ARPES. Propagation vector (α = 0°) of emitted electron and polarization vector of incident photon are on the YZ plane. The angle resolved measurements are made along *x* axis with |α| < 8°. The sample tilting angle is denoted by *θ*, which is rotation around the *x* axis. To simulate orientation disorder of the grains of HOPG, the sample is rotated around the *z* axis by the azimuthal angle *ϕ*.

**Figure 2:**
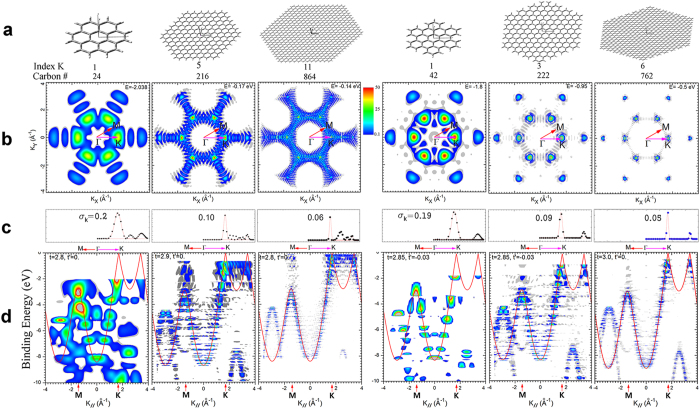
Calculated electronic structures of hexagonally symmetric PAHs with two different edge structures probed by a p-polarized 70 eV photon. (**a**) Structures of the molecules with various carbon numbers and edge structures. (**b**) k-space PES intensity map of HOMO. (**c**) Dots: Momentum distribution of HOMO along ΓK, which is indicated in (**b**). Red lines: Gaussian distribution curves which is obtained from curve fitting of the dots. Inset: σ_k_ is a standard deviation of momentum. The value is obtained from convolution using Gaussian distribution curves centered at the *K* point. (**d**) Calculated band structures are plotted along ΓK and ΓM. Colored contour map: Intensity of simulated photoemission. Red lines: Curves obtained from curve fitting using equation (1) and (2). Inset: *t* and *t*’ are the values of the parameters used to perform curve fitting.

**Figure 3:**
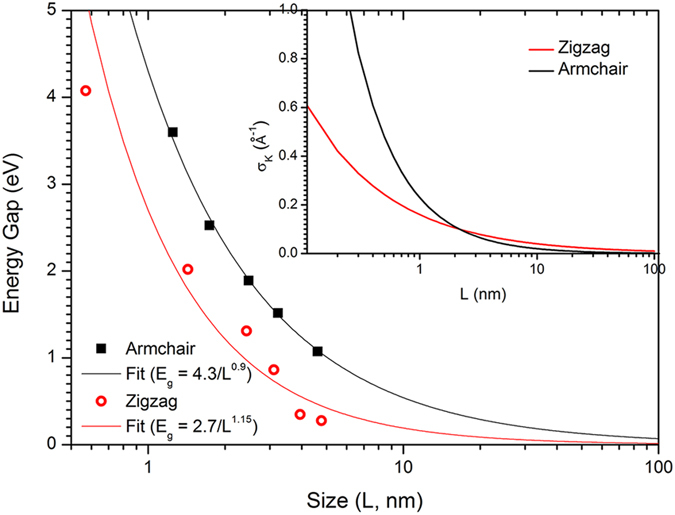
Quantum confinement effect as a function of the size of PAH. Energy gap and standard deviation of momentum (inset) are represented as a function of the size of PAH.

**Figure 4:**
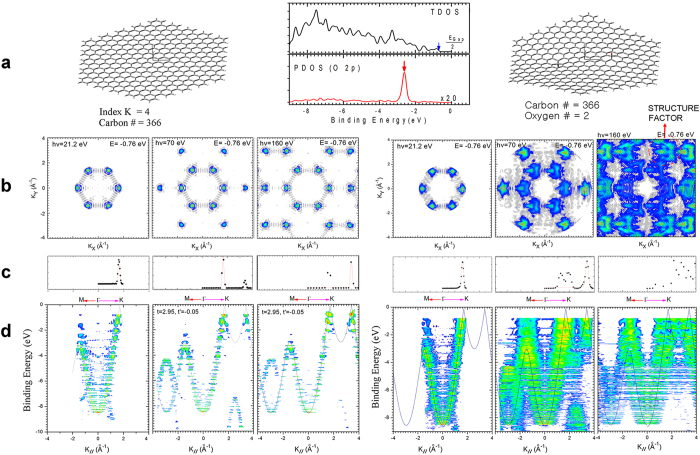
Calculated ARPES of a pristine PAH (left panels) and a chemically-ornamented molecule in which two epoxides are adsorbed on the PAH at the specified locations (right panels). (**a**) Geometry of each molecule; middle panel: total density of states (TDOS) and projected density of states (PDOS) of O2p. (**b**) k-space PES intensity map of HOMO for three different photon energies. (**c**) Momentum distribution curve of HOMO along ΓK. (**d**) Calculated band structures are plotted along ΓK and ΓM for three different photon energies.

**Figure 5:**
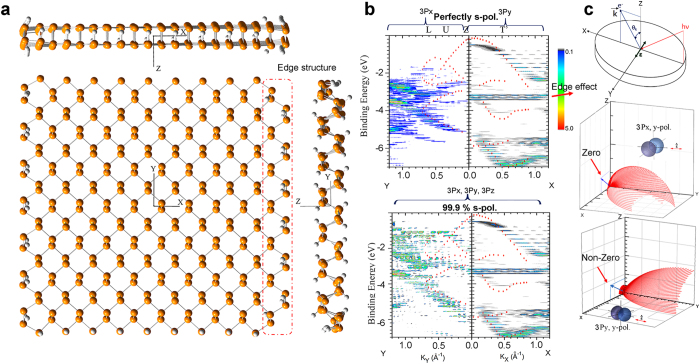
Calculated ARPES of BP. (**a**) Optimized geometry of a black phosphorus molecule with phosphine atom numbers of 278. Edge located phosphine atoms are hydrogen-terminated appropriately. (**b)** Calculated ARPES along *x* and *y* directions. Red dots: experimental results of the ref. 26. (**c)** Top panel: definition of photon polarization and electron measurement geometry. The middle and bottom panels show the PES cross sections from the 3p_x_ and 3p_y_ orbitals, respectively, when the *y*-polarized photon strikes the sample.
