# Supplementary material for: Understanding the Unique Electronic Properties of Nano Structures Using Photoemission Theory
Source: Sci Rep. 2015 Dec 4;5:17834. doi: 10.1038/srep17834 (PMC4669468; doi:10.1038/srep17834)
Supplement: Supplementary Information [file srep17834-s1.pdf]

[Supplementary Information]

Understanding the Unique Electronic Properties of Nano Structures Using Photoemission Theory

Soonnam Kwon[1] and Won Kook Choi*[2]*

[1] Beamline Division Group of PAL-XFEL Project Headquarters, Pohang university of science and technology, 77 Cheongam-Ro, Nam-Gu, Pohang, Gyeongbuk,Korea. 790-784

[2] Materials and Life Science Research Division, Korea Institute of Science and Technology (KIST), Hwarangno 14-gil 5, Sungbuk Gu, Seoul, Korea 136-791[*] Email address: snkwon@postech.ac.kr, and wkchoi@kist.re.kr

KEYWORDS: Nano graphene, Black phosphorous, ARPES, Photoemission theory, Density functional theory (DFT)

A. Fourier transform of the initial-state wave function

An initial eigen-function of a molecule can be expressed by using linear combination of atomic orbitals (LCAO) centered at each atomic position, $\boldsymbol{R}_{\alpha}$.

$$\Psi_i(\vec{r}) = \sum\sum C_{\alpha,nlm}\phi_{\alpha,nlm}(\boldsymbol{r} - \boldsymbol{R}_{\alpha}) \tag{S1}$$

If we take Fourier transform over the both sides of equation (S1) from real geometric space *r* into reciprocal space *k*,

$$\psi_i(\vec{k}) = \int d^3r\, e^{-i\vec{k}\cdot\vec{r}}\psi_i(\vec{r}) = \int d^3r\, e^{-i\vec{k}\cdot\vec{r}}\sum\sum C_{\alpha,nlm}\phi_{\alpha,nlm}(\boldsymbol{r} - \boldsymbol{R}_{\alpha})$$

$$= \sum\sum C_{\alpha,nlm}\, e^{-i\vec{k}\cdot\overrightarrow{R_\alpha}} \int d^3(\boldsymbol{r} - \boldsymbol{R}_{\alpha})\, e^{-i\vec{k}\cdot\overrightarrow{(r-R_\alpha)}}\,\phi_{\alpha,nlm}(\boldsymbol{r} - \boldsymbol{R}_{\alpha})$$

$$= \sum\sum C_{\alpha,nlm}\, e^{-i\vec{k}\cdot\overrightarrow{R_\alpha}}\,\phi_{\alpha,nlm}(\vec{k}) \tag{S2}$$

Podolsky, Pauling, and Gadzuk developed the mathematical tool for Fourier transformation of atomic orbitals.[S1, S2] For simplicity, if we consider only $2p_z$ atomic orbital, which is the case of π states of poly aromatic hydrocarbons (PAH), equation (S2) reduces to equation (S3) from the work of Gadzuk.[S2]

$$\psi_i(\vec{k}) = -4\pi \mathrm{i}\, Y_{10}(\theta_k, \varphi_k)\mathrm{f(k)} \sum C_{\alpha,210}\, e^{-i\vec{k}\cdot\overrightarrow{R_\alpha}}$$

$$= -4\pi \mathrm{i} \cos(\theta_k)\mathrm{f(k)} \sum C_{\alpha,210}\, e^{-i\vec{k}\cdot\overrightarrow{R_\alpha}} = -4\pi \mathrm{i}\, \frac{\sqrt{\mathrm{k}^2 - \mathrm{k}_{//}{}^2}}{\mathrm{k}}\mathrm{f(k)} \sum C_{\alpha,210}\, e^{-i\vec{k}\cdot\overrightarrow{R_\alpha}} \tag{S3}$$

where f(k) is a function of absolute value of k, and θ_k, φ_k are polar and azimuthal angles of vector $\boldsymbol{k}$.

B. Plane wave approximation to the final state wave function of photo-emitted electrons

$$\mathrm{I}_{\psi_i}\left(\widehat{\boldsymbol{k_f}}, \mathrm{E}_{k_f}\right) = \left|\ \langle \psi_{k_f} | \overrightarrow{\mathbf{A_0}} \cdot \vec{\mathbf{p}} | \Psi_i \rangle \right|^2 \delta(E_{\Psi_{kf}} - hv - E_{\Psi_i}) \quad \text{(S4)}$$

Equation (S4) denotes the photoelectron emission intensity at measured angle and energy of $\boldsymbol{k_f}$, and E_{kf}, respectively, from an initial state of ψ_i, where $E_{kf} = (h/2\pi)^2 k^2{}_f/2m_e$. If the final state wave function ψ_f is approximated to a plane wave,

$$\langle \boldsymbol{e^{-i\vec{k}_f \cdot \vec{r}}} | \overrightarrow{\mathbf{A_0}} \cdot \vec{\mathbf{p}} | \Psi_i \rangle = \overrightarrow{\mathbf{A_0}} \cdot \overrightarrow{\mathbf{k_f}}\ \langle \boldsymbol{e^{-i\vec{k}_f \cdot \vec{r}}} | \Psi_i \rangle = \overrightarrow{\mathbf{A_0}} \cdot \overrightarrow{\mathbf{k_f}}\ \Psi_i(\overrightarrow{k_f}) \quad \text{(S5)}$$

From equation (S4) and (S5) we obtain the following.

$$\mathrm{I}_{\psi_i}\left(\widehat{\boldsymbol{k_f}}, \mathrm{E}_{k_f}\right) = \left|\overrightarrow{\mathbf{A_0}} \cdot \overrightarrow{\mathbf{k_f}}\ \Psi_i(\overrightarrow{k_f})\right|^2 \delta(E_{\Psi_{kf}} - hv - E_{\Psi_i}) \quad \text{(S6)}$$

Substituting equation (S3), we get the following for any k_f satisfying $E_{\Psi_{kf}} = E_{\Psi_i} + hv$,

$$\mathrm{I}_{\psi_i}\left(\widehat{\boldsymbol{k_f}}, \mathrm{E}_{k_f}\right) = \left|\overrightarrow{\mathbf{A_0}} \cdot \overrightarrow{\mathbf{k_f}}\ \right|^2 \left| \frac{\sqrt{\mathrm{k}_f{}^2 - \mathrm{k}_{//}{}^2}}{\mathrm{k}_f} \mathrm{f}(\mathrm{k}_f) \sum C_{\alpha,210}\, e^{-i\vec{k}_f \cdot \vec{R}_\alpha} \right|^2 \quad \text{(S7)}$$

However, this expression does not contain any information related to the quantum mechanical transition probability from initial to final state, which should include dipole selection rule. This equation just reflects the special distribution of molecular orbital of the initial state modulated with the incident light polarization effects on the final state electrons.[S3]

Even though this simple approach can give useful visual guidance to the experimental results, the experimental result can be significantly different from these simple results, in most of the situations. Therefore, for more realistic correlation between experimental and theoretical results, the exact interaction between photon and electron in the atomic or molecular situation should be considered.[S4, S5]

C. Photoemission intensity from a molecule obtained by quantum mechanical calculation using IAC approximation

The PES cross section of the *n*th Kohn-Sham (K.-S.) energy level, which corresponds to ψ_i in equation (S7), can be defined by

$$\mathbf{I_n}(\vec{\mathbf{R}}, \boldsymbol{E_{kin}}) \propto \left|\mathbf{A^n_{tot}}(\vec{\mathbf{R}}, \boldsymbol{E_{kin}})\right|, \tag{S8}$$

$$\mathbf{A_n}(\vec{\mathrm{R}}, \boldsymbol{E_{kin}}) = \frac{\mathbf{e^{ikR}}}{\mathbf{R}} \sum_{\mathbf{a}} \mathbf{C_{na}} \mathbf{e^{i\vec{k}\cdot\vec{R}_a}} \sum_{\mathbf{l,m}} \mathbf{M^m_{l,a} y^m_l}(\hat{\mathbf{R}}) \tag{S9}$$

where $\vec{\mathbf{R}}$, and $\vec{\mathbf{R}}_\mathbf{a}$, represent position vectors of detector and the *a*th atom in a molecule with respect to origin, respectively. E_{kin}, and $\vec{\mathbf{k}}$ denote kinetic energy and the momentum vector of a photo-emitted electron, respectively.[S6] $\sum_{\mathbf{l,m}} \mathbf{M^m_{l,a} y^m_l}(\hat{\mathbf{R}})$ is an atomic factor that represents the PES probability from the *a*th atom, and can be written as,

$$\mathbf{M^m_{l,a} y^m_l}(\hat{\mathbf{R}}) \propto \langle \boldsymbol{\phi}_{\mathbf{Ekin},\vec{\mathbf{k}}} | \hat{\boldsymbol{\varepsilon}} \cdot \vec{\mathbf{r}} | \boldsymbol{\phi}^{\mathbf{a}}{}_{\mathbf{qlm}} \rangle = \sqrt{4\pi R_s^2 E_{kin}}\, \hat{\boldsymbol{\varepsilon}} \cdot \hat{\boldsymbol{k}} \quad \text{for s orbital,}$$

$$(-\mathbf{i})^{\mathbf{l-1}}\left\{\mathbf{e}^{\mathbf{i}\boldsymbol{\delta}^{a}_{\mathbf{l-1}}}\mathbf{X_{l-1,m}} + \mathbf{e}^{\mathbf{i}\boldsymbol{\delta}^{a}_{\mathbf{l+1}}}\mathbf{X_{l+1,m}}\right\} \quad \text{for p and d orbital} \tag{S10}$$

where, R_S is the radial dipole matrix element of s orbital, $\boldsymbol{\delta}^{\mathbf{a}}_{\mathbf{l\pm1}}$ is the overall phase shift of the atom indexed by "a", and $\mathbf{X_{l\pm1,m}}$ are the functions defined in reference S7.

C_{na} represent the coefficients of *a*th atom to the nth molecular orbital wave function, which is obtained using pbeh1pbe/sto-3g level of theory, in which sto-3g uses minimum atomic orbitals.

D. Angle resolved photoemission simulation

Based on equation (S7) or (S8), the photoemission yields can be obtained as a function of momentum vectors of emitted electrons. The momentum vectors include the information on the kinetic energy and the direction of emitted electrons, which are polar and azimuthal angles.

$$E_B = h\nu - E_{kin} - \phi \tag{S11}$$

$$K_x = \frac{\sqrt{2m_eE_{kin}}}{2\pi h}\sin\theta\cos\varphi,\ K_y = \frac{\sqrt{2m_eE_{kin}}}{2\pi h}\sin\theta \tag{S12}$$

Where hv is the incident photon energy and Φ is the work function of the system. Using equation S11 and S12, binding energy of an electron with momentum K_x and K_y can be easily calculated if the signal intensity is measured for each detector geometry (θ and φ) and kinetic energy of the electron. After conducting simulated experiments using equation (S7) or (S8), band structure as a function of surface parallel reciprocal momentum K_x and K_y can be obtained. The band mapping along high symmetry lines can be obtained by cutting the above complete band structure into vertical cross section with specific azimuthal angle corresponding to the high symmetry line. And the ARPES intensity mapping versus two dimensional surface parallel momenta is obtained by horizontal cross section of the complete band structure with a specified energy level. The K.-S. energy scale is compressed to compensate for the approximation of electronic relaxation and correlation effects inherent in the DFT functional used. The binding energy was scaled so as to coincide with the experiment as close as possible. Then, the overall energies were shifted appropriately.

Fourier transform of initial state wave function into k-space reflects the initial state and can be related to the final state within the assumption of plane wave final state, which can also be associated to the PES intensity as shown in equation (S7).

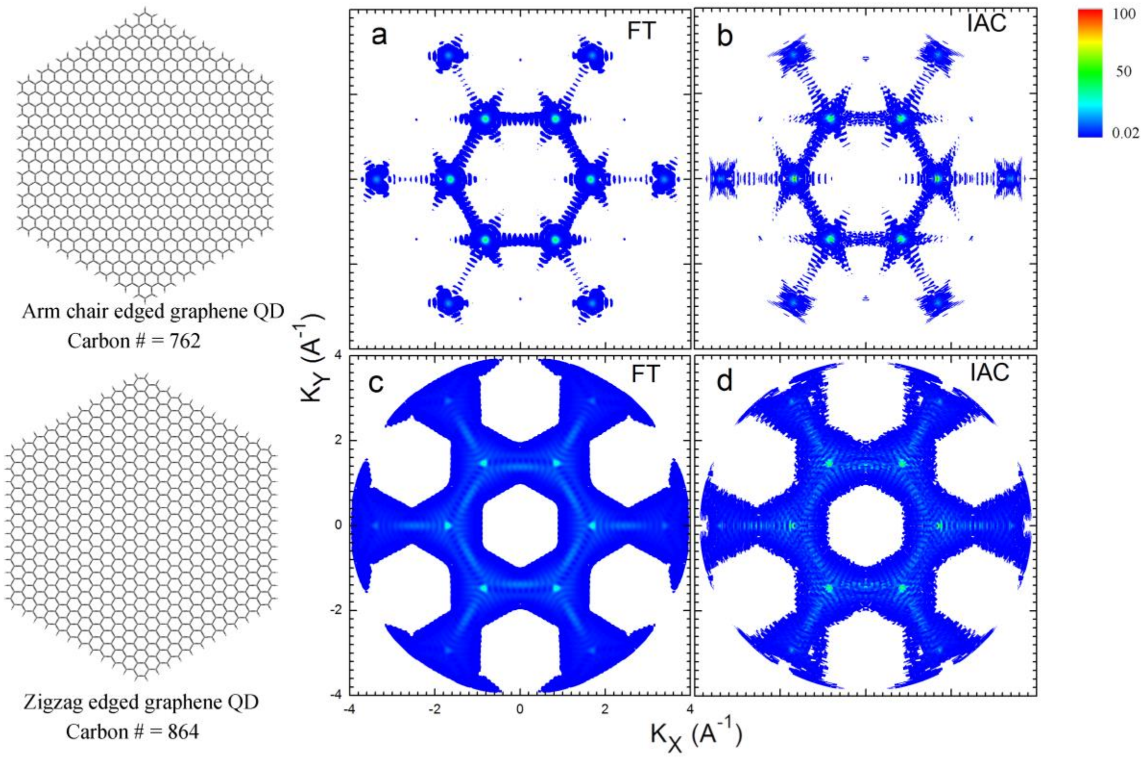

Figure. S1. **Comparison between PW approximation and IAC for hexagonally symmetric PAHs with two different type of edge structures. a and c,** PW with Fourier transformation (FT) of initial stat. **b** and **d,** IAC approximation. IAC results were calculated using excitation photon energy of 70 eV.

The same PES intensity can also be obtained using PES simulation equipped with the formalism exploiting the IAC approximation as shown in equation (S9). To show the validity and the usefulness of this method, the IAC simulated PES intensity on the well-known graphene nano structures is compared to that using Fourier transform and plane wave final states. Figure S1 shows

two different types of nanoscale graphenes, which are armchair and zigzag edged graphenes respectively. The two different approaches using FT and IAC produce almost the same results, which had been predicted by the reports of Puchnig *et al*.[S3] The most prominent feature is the different contribution of the edge induced effects on the original electronic structure. The zigzag edge induced features are obviously seen in Figure S1c and d, which correspond to the unique features interconnecting K points near the Fermi surface. On the contrary, armchair edged graphene exhibits significantly low surface state intensity at the highest occupied energy states, as shown in Figure S1a and b.

E. ARPES experiments on HOPG and comparison to the photoemission simulation

ARPES experiments were conducted under UHV of 2.0×10^{-10} Torr at the 8A2 undulator beam line of the Pohang Accelerator Laboratory (PAL) in Korea using a high-resolution electron analyzer, VG Scienta SES 2002 with a 2D-CCD detector. Spectra of the valence bands were obtained using linearly polarized photons with energies ranging from 100 eV to 400 eV. All the experiments were performed at room temperature. The HOPG crystal was cleaved outside the vacuum chamber using scotch tape and introduced into the chamber immediately. The sample was degassed at 400°C for more than 5 hours to remove any adsorbed molecules. The cleanness of the sample was confirmed by the measurements of the core levels of nitrogen, oxygen, and carbon.

To simulate HOPG as a molecule, an armchair edged hexagonally PAH with carbon numbers of 762 is used as shown in Figure 2. All the calculations were performed on the platform of Gaussian 09 package.[S8] The geometry was fully optimized using PBEh1PBE/sto-3g level of theory. The K.-S. energy scale is compressed to compensate for the approximation of electronic relaxation and

correlation effects. In our calculation, we chose a compensation factor of 0.972. This value was obtained by scaling the binding energy of the calculated band structure to coincide with the experiment. And the overall energies are shifted so as for the midpoint between HOMO and LUMO to be zero. Figure S2 shows ARPES spectra using various photon energies with the experimental geometry shown in Figure 1c. The angle resolved electron measurements are made along the x-axis and indicated as α's. To simulate the randomly oriented graphite domains of HOPG, the total PES intensities are averaged over those of PAHs with 360 equally spaced azimuthal angles, ϕ's.

Figure S3a illustrates the ARPES simulation results using 100 eV photon and large acceptance angle (α = ± 30°). The simulation took into account of the disorder in azimuthal orientation of graphite grains by averaging the ARPES simulation over all possible orientation. The simulation show great resemblance with the experiments of this study and the others.[S9, S10]

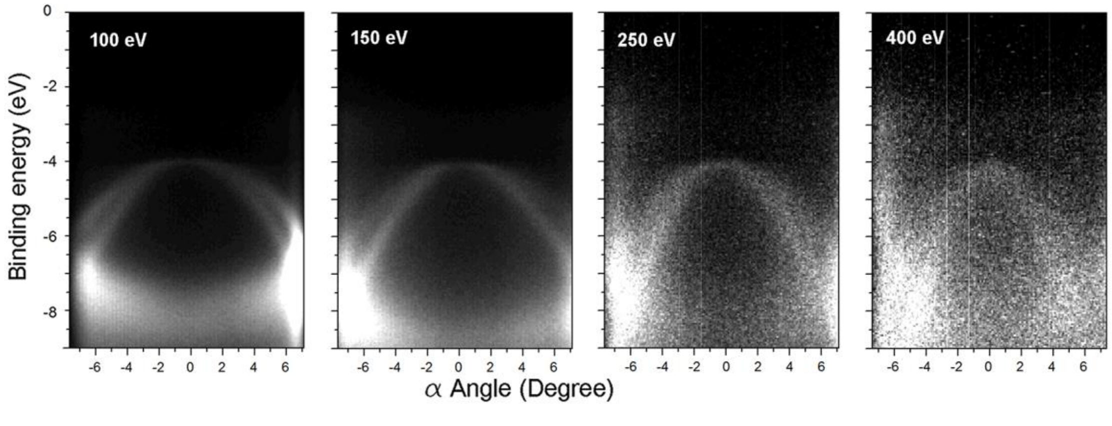

Figure. S2. **Experimental ARPES data using various photon energy and tilt angles (θ = 0)**

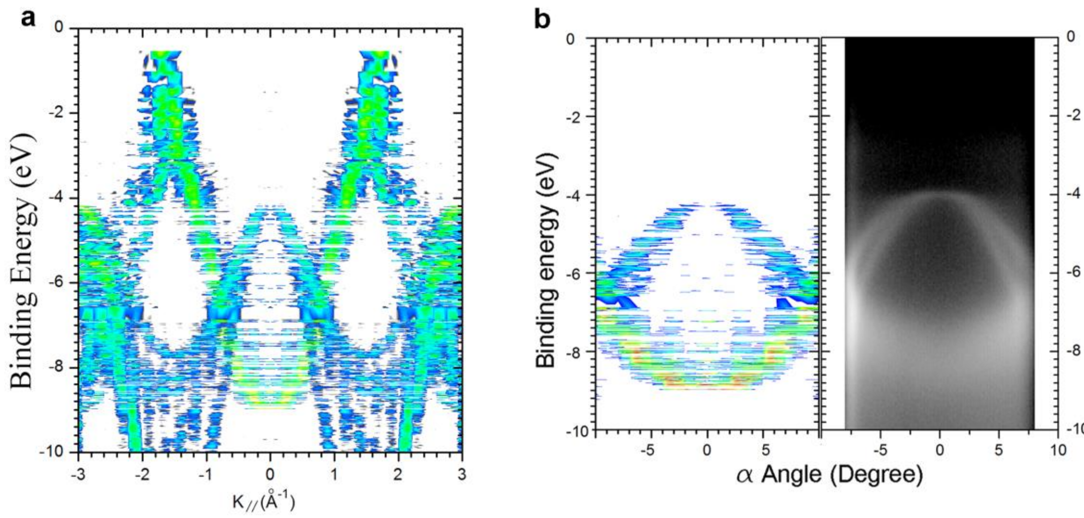

Figure. S3. **Comparison between calculated and experimental ARPES. a,** ARPES simulation of HOPG as a function of surface parallel momentum using photon energy of 100 eV. **b,** Left panel: The same with (**a**), but the x-axis is the acceptance angle in the range of ± 10˚. Right panel: Experimental data which is the same with Figure 1b and c except the tilt angle θ = 0.

REFERENCES

S1. Podolsky, B. & Pauling, L. *Phys. Rev.* **34**, 109 (1929).

S2. Gadzuk, J. W. *Phys. Rev.* B 10, 5030 (1974).

S3. Puschnig, P., Koller, G., Draxl, C. & Ramsey, M. G. The structure of molecular orbitals investigated by angle-resolved photoemission: *Small Organic Molecules on Surfaces*, edited by Sitter, H., Draxl, C. & Ramsey, M. (Springer-Verlag, Berlin, Heidelberg, 2013).

S4. Fujikawa, T. *J. Phys. Soc. Jpn* **50**, 1321 (1980).

S5. Richardson, N. V. *Chem. Phys. Lett.* **102**, 390 (1983).

S6. Grobman, W. D. *Physical Review* B **17**, 4573 (1978).

S7. Goldberg, S. M., Fadley, C. S. & Kono, S. *J. Elect. Spec. Rel. Phen.* **21**, 285 (1981).

S8. Frisch, M. J., Trucks, G. W. & Schlegel, H. B. *et al.*, GAUSSIAN 09, Revision A.02, Gaussian, Inc., Wallingford, CT, 2009.

S9. Mahatha, S. K. & Meon, K. S. R. *Phys. Rev.* B 84, 113106 (2011).

S10. Zhou, S. Y. *et al. Phys. Rev.* B **71**, 161403 (2005).
